# Editorial: Sulphur Metabolism of Fungi - Implications for Virulence and Opportunities for Therapy

**DOI:** 10.3389/fmicb.2020.583689

**Published:** 2020-10-08

**Authors:** Jorge Amich, Sven Krappmann, Anand Kumar Bachhawat

**Affiliations:** ^1^Manchester Fungal Infection Group (MFIG), Faculty of Biology, Medicine and Health, University of Manchester, Manchester, United Kingdom; ^2^Mikrobiologisches Institut - Klinische Mikrobiologie, Immunologie und Hygiene, Universitätsklinikum Erlangen and Friedrich-Alexander-Universität Erlangen-Nürnberg, Erlangen, Germany; ^3^Indian Institute of Science Education and Research (IISER), Chandigarh, India

**Keywords:** sulfur metabolism of fungi, pathogenic fungi, implications for virulence, opportunities for therapy, fungal primary metabolism

Fungal infections pose a global and constant threat, especially to immunocompromised individuals. This unresolved situation is aggravated by diagnostic as well as therapeutic shortcomings. Metabolism is a fundamental aspect of fungal physiology and vitality and has been recognized to be crucial for pathogenicity. In this respect, sulfur metabolism is of special interest as it comprises essential metabolic routes that are not well-conserved in host cells and, therefore, provide prospects for novel antifungal targets and therapies. Exogenous sulfur is assimilated and incorporated into biosynthetic pathways through the trans-sulfuration pathway, which constitutes the core of fungal sulfur metabolism and yields proteinogenic amino acids methionine and cysteine. From there, sulfur derives in a variety of different molecules, that include sulfur containing vitamins and co-factors such as biotin, thiamine, pantothenic acid, coenzyme A, and the antioxidant glutathione, thereby extending the involvement of sulfur metabolism to a plethora of cellular processes. Some of these molecules have already been directly and indirectly implicated in fungal virulence, further supporting sulfur metabolism as an area of interest in the search for novel treatments.

The articles in this Research Topic reflect the breadth of processes for which sulfur metabolism is relevant in the context of fungal pathogenicity, highlighting the many opportunities for the identification of novel, promising pan-fungal targets. It is established that fungal redox homeostasis strongly relies on sulfur-containing factors, such as glutathione. Extending these past findings, Binder et al. demonstrate that thioredoxin constitutes another system based on sulfur that contributes to *A. fumigatus* oxidative stress resistance and virulence. In pathogenic fungi, the transcriptional regulation of sulfur metabolism is controlled by the Cys3/MetR transcriptional regulator. In this collection of articles, Martho et al. show that in *Cryptococcus neoformans* the phosphatase Gpp2 modulates Cys3 activity in response to osmotic stress, connecting oxidative stress, sulfur uptake, and the osmotic stress response, all of which determine fungal virulence. Sulfur is also required for the biosynthesis of S-adenosylmethionine (SAM), the key source of methyl groups in cellular transmethylation reactions. Correspondingly, Traynor et al. review the sulfur assimilation route and its incorporation in *A. fumigatus* into SAM, the S-containing mycotoxin gliotoxin, and ergothioneine, a metabolite involved in redox homeostasis. The authors describe how gliotoxin balances the homeostatic regulation of SAM, how it interplays with ergothioneine biosynthesis and also connects with zinc metabolism, all essential processes for fungal virulence (Traynor et al.). In this collection of articles, Walvekar and Laxman summarize and discuss how methionine serves as a signal to activate anabolism and connects sulfur metabolism to NADPH homeostasis. Volatile sulfur-compounds (VSCs) are particularly relevant when considering the involvement of S-containing molecules in signaling processes. VSCs can exert long-distance intra- and inter- species interactions, and Scott et al. demonstrate that *Pseudomonas aeruginosa*-derived VSCs can stimulate fungal growth *in vivo*, which causes an increment in pathogen burdens and higher mortality in the *Galleria mellonella* infection model of aspergillosis.

This Research Topic underlines the broad range of processes for which sulfur metabolism is important in the context of the virulence of human fungal pathogens. Sulfur metabolism holds great potential to identify novel fungal targets and, therefore, efforts should be made to understand sulfur metabolism and exploit it in order to develop unique strategies of antifungal therapy.

## Author Contributions

All authors listed have made a substantial, direct and intellectual contribution to the work, and approved it for publication.

### Conflict of Interest

The authors declare that the research was conducted in the absence of any commercial or financial relationships that could be construed as a potential conflict of interest.

